# Automated identification of innocent Still's murmur using a convolutional neural network

**DOI:** 10.3389/fped.2022.923956

**Published:** 2022-09-21

**Authors:** Raj Shekhar, Ganesh Vanama, Titus John, James Issac, Youness Arjoune, Robin W. Doroshow

**Affiliations:** ^1^Sheikh Zayed Institute for Pediatric Surgical Innovation, Children's National Hospital, Washington, DC, United States; ^2^AusculTech Dx, Silver Spring, MD, United States; ^3^Children's National Heart Institute, Children's National Hospital, Washington, DC, United States

**Keywords:** Still's murmur, innocent heart murmur, convolutional neural network, automated identification, artificial intelligence

## Abstract

**Background:**

Still's murmur is the most prevalent innocent heart murmur of childhood. Auscultation is the primary clinical tool to identify this murmur as innocent. Whereas pediatric cardiologists routinely perform this task, primary care providers are less successful in distinguishing Still's murmur from the murmurs of true heart disease. This results in a large number of children with a Still's murmur being referred to pediatric cardiologists.

**Objectives:**

To develop a computer algorithm that can aid primary care providers to identify the innocent Still's murmur at the point of care, to substantially decrease over-referral.

**Methods:**

The study included Still's murmurs, pathological murmurs, other innocent murmurs, and normal (i.e., non-murmur) heart sounds of 1,473 pediatric patients recorded using a commercial electronic stethoscope. The recordings with accompanying clinical diagnoses provided by a pediatric cardiologist were used to train and test the convolutional neural network-based algorithm.

**Results:**

A comparative analysis showed that the algorithm using only the murmur sounds recorded at the lower left sternal border achieved the highest accuracy. The developed algorithm identified Still's murmur with 90.0% sensitivity and 98.3% specificity for the default decision threshold. The area under the receiver operating characteristic curve was 0.943.

**Conclusions:**

Still's murmur can be identified with high accuracy with the algorithm we developed. Using this approach, the algorithm could help to reduce the rate of unnecessary pediatric cardiologist referrals and use of echocardiography for a common benign finding.

## Introduction

Still's murmur is the most prevalent innocent heart murmur of childhood (1). It is a low-pitched, musical, early-to-mid systolic murmur primarily heard at the lower left sternal border (LLSB) (2–6). It is ~50 times more common than murmurs of congenital heart disease (7). It does not reflect any abnormality and does not require any treatment.

Auscultation is the primary clinical tool to identify this murmur as innocent (8, 9). Whereas pediatric cardiologists routinely perform this task, primary care providers (PCPs) have significantly less success in distinguishing Still's murmur from the murmurs of true heart disease (9, 10). Their identification accuracy is higher (although still poor) for pathological murmurs (41%) and other innocent murmurs (45%) (11). Multiple studies report that only cardiologists can reliably distinguish different murmur types with a stethoscope (3, 4, 11–14). Currently, when a PCP hears a Still's murmur in a child, they will consider referring the child for expert consultation, even when symptoms are absent (15). Uneven referral patterns suggest that not all children with Still's murmur are referred, and that PCPs vary greatly in deciding whom to refer (14). Those children who are referred undergo consultations and associated tests which may be costly and waste healthcare resources, and are a source of avoidable anxiety for children and families (16, 17).

Most heart murmurs, including Still's murmur, are characterized by several features including the location on the chest where they are heard and the timing of the murmur signal within the cardiac cycle. A cardiac cycle is marked by the S1 (onset of systole) and S2 (onset of diastole) sounds. Still's murmur's signal has a distinctive diamond-shaped envelope of a relatively pure low frequency that occurs shortly after S1. These stereotypical features allow for robust classification of Still's murmur (18). Pathological murmurs, conversely, can vary widely in their frequency content and timing, depending on the lesion. From a classification standpoint, this large variation renders it very challenging to correctly identify pathological murmurs automatically by specific diagnosis.

Computer-aided auscultation is an area of active research (19–21). Classification methods include support vector machine (SVM), artificial neural network (ANN), hidden Markov model, and k-nearest neighbor, among others (19, 20). More recently, with the emergence of deep learning and growing availability of heart sound data, new classification approaches employing convolutional neural networks (CNNs) are being proposed (22, 23).

Multiple prior studies have attempted to make a specific diagnosis for each pathological murmur (24–29). This task is complex and of limited clinical value. A child with a pathological murmur of any etiology needs to be seen by a pediatric cardiologist who will employ detailed echocardiography and other tests to confirm the diagnosis before initiating treatment (3). The focus of our work has been automated algorithmic identification of Still's murmur, specifically. We have reported an algorithm for segmenting cardiac cycles and identifying Still's murmur using traditional machine learning (18, 30). With the acquisition of additional data, we describe here a deep-learning algorithm based on a CNN framework. As in our previous work, the algorithm performs a binary classification to distinguish Still's murmur from all other pediatric heart murmurs.

## Materials and methods

### Heart sound recordings

For algorithm development, we used the Murmur Library, a database of deidentified recordings of pediatric heart sounds and murmurs compiled by one of the authors (RWD), a board-certified pediatric cardiologist with >40 years of experience. The recordings were obtained from supine pediatric subjects, 2–17 years of age, with parental consent (31), using a commercially available electronic stethoscope (Model 4100, 3M Littmann, St. Paul, MN). For each murmur recording, the definitive clinical diagnosis was documented. All suspected pathological murmurs were confirmed by echocardiography. For Still's and other innocent murmurs, we used the pediatric cardiologist's diagnosis, including the findings of an echocardiogram if performed. The study was approved by the Children's National Hospital Institutional Review Board.

We identified 1,473 applicable recordings in the Murmur Library and binned them into three classes: no murmur, Still's murmur, and murmurs other than Still's (see Table 1). The last class included predominantly pathological murmurs and some innocent murmurs that were not Still's murmurs. A detailed breakdown of murmur types for this class is provided in Table 2. The typical length of a recording was 7–15 s, and they came from all four typical chest locations for cardiac auscultation: right upper sternal border, left upper sternal border, LLSB, and apex. Because Still's murmur is best heard at the LLSB, we also formed a subset of LLSB-only recordings. This allowed us to investigate classification strategies based on the recording location.

**Table 1:** Number of heart sound recordings organized by clinical diagnosis and recording location used in the study.

**Recording location**	**No murmur**	**Still's murmurs**	**Murmurs other than Still's**	**Total**
All locations	321	270	882	1,473
LLSB	254	232	323	809

**Table 2:** Distribution of pathological and innocent murmurs other than Still's at all locations (*N* = 882).

Ventricular septal defect	296
Venous hum	102
Pulmonic stenosis	100
Patent ductus arteriosus	56
Aortic stenosis	46
Tricuspid regurgitation	40
Mitral regurgitation	38
Innocent right ventricular outflow murmur	37
Atrial septal defect	35
Subaortic stenosis	26
Pulmonic regurgitation	21
Aortic insufficiency	12
Shunt	10
Pulmonary artery branch stenosis	9
Tetralogy of Fallot	5
Multiple aortopulmonary collaterals	4
Pulmonic stenosis and atrial septal defect	4
Supravalvular aortic stenosis	4
Physiologic peripheral pulmonary artery stenosis	3
Pulmonic stenosis and tetralogy of Fallot	3
Pulmonary artery branch stenosis	3
Mitral stenosis	3
Tricuspid stenosis	3
Aortic insufficiency and VSD	2
Aortic stenosis and aortic insufficiency	2
Arteriovenous malformation	2
Coronary artery fistula	2
Hypertrophic obstructive cardiomyopathy	2
Interrupted aortic arch complex	2
Atrioventricular canal	1
Aortic valve replacement	1
Double-chambered right ventricle	1
Left ventricular assist device	1
Left ventricular outflow tract	1
Mitral valve prolapse	1
Pulmonic stenosis and insufficiency	1
Subaortic stenosis with right bundle branch block	1
Subpulmonic stenosis	1
Supravalvular pulmonic stenosis	1

### Algorithm overview

**Figure 1:**
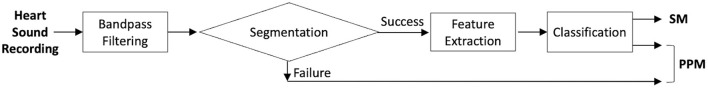
Algorithm flowchart.

The overall goal of the algorithm was to perform a binary classification on heart sound recordings, with two possible outputs: Still's murmur (SM) or potentially pathological mumur (PPM). We tested four classification strategies using different subsets of the data, using the same overall structure of the algorithm (see Figure 1). Each single cardiac cycle was individually classified. Consequently, every recording went through preprocessing to identify and prepare individual cycles for classification.

A heart sound recording was first bandpass filtered to remove any acquisition noise and to focus on frequencies of interest. The bandpass filter was a 4th-order Butterworth filter with a passband of 40–500 Hz. The filter design was informed by the fact that the normal heart sound frequencies range from 20 to 200 Hz. The frequency of Still's murmur varies between 90 and 170 Hz, and pathological murmurs are in the range of 80–500 Hz (32). The next step was the application of a previously reported segmentation technique that identified S1 and S2 sound lobes in the recording and used them to identify individual cardiac cycles (18, 30). We defined a cardiac cycle as the period between the onsets of two consecutive S1 sounds.

A heart sound recording can either be successfully segmented or fail segmentation. If a recording is successfully segmented, it proceeds to the next stage of the algorithm comprising feature extraction and classification. If a recording fails segmentation, there is a high probability that S1 and S2 sounds are indistinguishable from the surrounding murmur and heart sound signals and cannot be identified. This happens chiefly for holosystolic or continuous murmurs, which at the LLSB are pathological. Recordings with excessive acquisition noise that cannot be removed by the bandpass filter can also cause segmentation failure. When segmentation fails, we directly classify the recording as PPM because segmentation rarely fails for Still's murmur recordings. Note that designating a recording of a suspected pathological murmur or suboptimal sound quality as PPM follows the status quo and no harm is done. In these cases, the provider will be expected to use the current clinical standard to determine the next course of action.

The classification was performed using a CNN. The feature extraction step was to prepare each cardiac cycle for input to the CNN, and required transforming each heart-cycle signal to a spectral image (i.e., a spectrogram). Segmented cardiac cycles were converted into grayscale spectrograms that varied in length due to their varying durations. To generate fixed-sized spectrograms irrespective of the cycle length, the original spectrograms were resampled to a 55 × 129 grid size and then converted to the decibel scale and normalized. The normalization was further necessary to reduce the size of the input image to the CNN and thus have a small architecture that avoided overfitting and the need for additional training data.

### CNN classification

For our binary classification problem—SM or PPM—we developed a five-layer CNN. The CNN architecture consisted of an input layer, three convolution layers, two hidden layers, and an output layer. The input to the CNN was a spectrogram as described. Each of the three convolution layers consisted of a two-dimensional convolution “ReLU” activation function, max pooling, and padding as “same.” Following the convolution layers, the model had two fully connected layers, each of which had 64 units with “ReLU” activation function. The output layer was a single-neuron layer with sigmoid as an activation function. The loss function was a modified cross-entropy function to deal with the unbalanced data set. The optimizer was simple momentum optimizer, and the learning rate was set to 0.9 with an exponential decay.

The output of the CNN varied between 0 and 1 which we associated with the probability of an input cardiac cycle with Still's murmur. A value closer to 1 meant that the cycle had a high probability of being a Still's murmur, whereas a value closer to 0 meant that it was something other than a Still's murmur. The default threshold for a cardiac cycle to be labeled as either SM or PPM was 0.5. The threshold was also varied to create a receiver operating characteristic (ROC) curve and calculate its area under the curve (AUC). The final classification of a recording depended on the majority decision from all cardiac cycles present in the recording. The CNN was developed and trained using TensorFlow (33) on an Nvidia GeForce RTX 2080 graphics computing unit with 12 GB memory.

### Training and validation set formation

**Figure 2:**
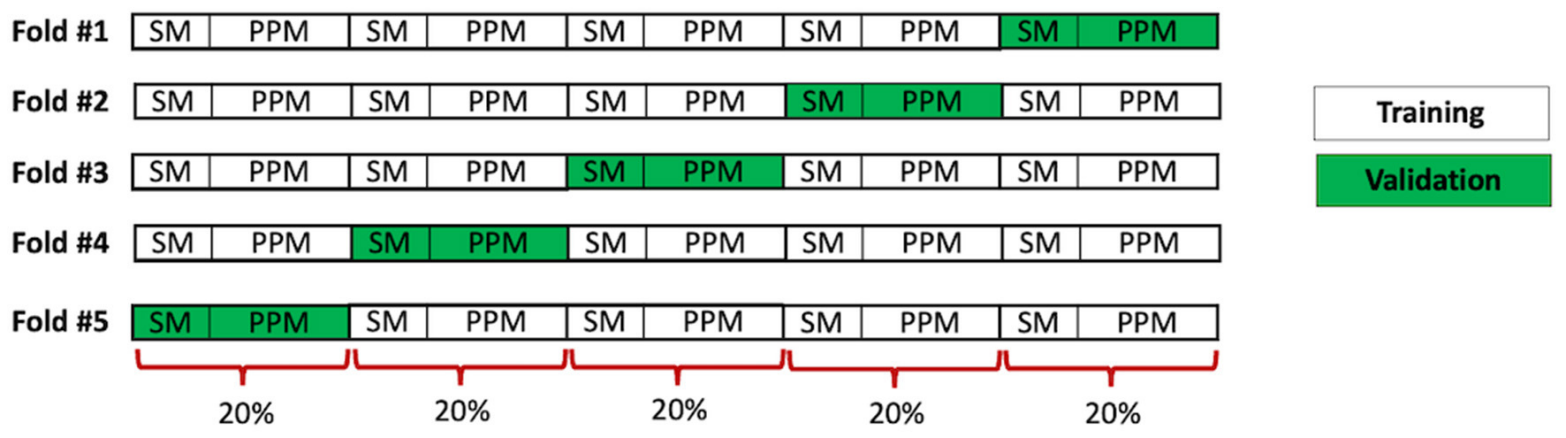
Schematic showing formation of training and validation sets for 5-fold cross validation.

We compared four different classification strategies that were designed to investigate the effects of recording location and of including normal (i.e., non-murmur) heart sound recordings in training and testing. This allowed training and testing of the algorithm with different subsets of the data. The process of forming the training and validation sets in each case followed the same procedure. Twenty percent of the Still's murmur recordings and 20% of other murmur recordings were randomly selected to form a validation set; the remaining 80% formed the training set. We further utilized a five-fold cross-validation strategy, whereby we created five sets of training and validation sets, with each validation set including a unique set of 20% of the Still's murmur and other murmur recordings. Thus, the CNN was trained five times using different training sets and tested five times as well for each classification strategy. The data preparation for this cross-validation procedure is shown schematically in Figure 2.

The time to train each CNN model was ~4 min. Recordings in both training and validation data sets went through the same processing steps outlined in Figure 1. The final performance figures were arrived at by aggregating the results of all five validation sets from the five folds. An advantage of cross-validation was that each recording was used once for testing the classifiers. The inference time for testing (i.e., classifying) a single recording was ~1.2 s.

### Test set formation

We found that the best performance was achieved when the algorithm was trained and tested using only murmur recordings (i.e., excluding non-murmur heart sound recordings) obtained at the LLSB (see Results below). Therefore, we further explored this strategy, forming a test set comprised of 60 Still's murmurs and 60 pathological murmurs from 120 unique patients that were recorded at the LLSB and had confirmatory echocardiograms. The distribution of pathological murmurs in this set mirrored their distribution in our database (see Table 3). The specific breakdown of the 60 pathological murmurs by diagnosis is provided in Table 4. The test set did not include any innocent murmurs other than Still's murmur. Such murmurs (e.g., venous hum) are generally not heard at the LLSB and represent a small minority of the innocent murmurs referred to our outpatient service. The training set was formed using the remaining data.

**Table 3:** Distribution of pathological and innocent murmurs other than Still's at LLSB (*N* = 323).

Ventricular septal defect	244
Tricuspid regurgitation	30
Pulmonic stenosis	11
Atrial septal defect	6
Aortic stenosis	6
Mitral regurgitation	5
Patent ductus arteriosus	4
Tetralogy of Fallot	3
Pulmonic regurgitation	3
Subaortic stenosis	2
Tricuspid stenosis	2
Aortic insufficiency	2
Aortic stenosis and aortic insufficiency	1
Pulmonic stenosis and regurgitation	1
Hypertrophic obstructive cardiomyopathy	1
Multiple aorticopulmonary collateral arteries	1
Supravalvular aortic stenosis	1

**Table 4:** Distribution of pathological murmurs in the test set (***N*** = 60).

Ventricular septal defect	41
Tricuspid regurgitation	5
Pulmonic stenosis	2
Atrial septal defect	1
Aortic stenosis	1
Mitral regurgitation	1
Patent ductus arteriosus	1
Tetralogy of Fallot	1
Pulmonic regurgitation	1
Tricuspid stenosis	1
Aortic insufficiency	1
Pulmonic stenosis and regurgitation	1
Hypertrophic obstructive cardiomyopathy	1
Multiple aorticopulmonary collateral arteries	1
Supravalvular aortic stenosis	1

## Results

By design, our algorithm performed a binary classification. In clinical terms, we defined the algorithm result as positive when its output was SM. The negative result, therefore, was the designation of a murmur as PPM. Following this convention, a ***true positive*** is a known Still's murmur correctly classified as SM, and a ***true negative*** occurs when the algorithm correctly identifies a recording known not to be Still's murmur as PPM. It follows that a ***false negative*** is a known Still's murmur wrongly designated as PPM, and a ***false positive*** is a murmur which is ***not*** Still's, wrongly classified as SM.

### Segmentation results

Because our classification is based on analysis of individual cardiac cycles, in Table 5, we present the result of segmentation and the number of available examples with which the CNN was trained and tested. Note that the recordings in which segmentation failed did not contribute to the cardiac cycle count.

**Table 5:** Number of cardiac cycles recorded, by location and diagnostic category.

**Recording location**	**No murmur**	**Still's murmurs**	**Murmurs other than Still's**	**Total**
All locations	2,476	2,544	7,377	12,397
LLSB	1,936	2,237	3,303	7,476

### Five-fold experiment

Table 6 lists the classification performance of the algorithm, using the default decision threshold of 0.5, under the four testing strategies. The algorithm performed better when the training and test data included only the LLSB recordings (right column). The best performance was achieved when training and testing of the algorithm was further constrained to only murmur recordings, both innocent and pathological but excluding normal, non-murmur recordings.

**Table 6:** Performance (sensitivity, specificity, accuracy) of the algorithm with five-fold cross-validation for the four Still's murmur identification strategies.

**Testing strategy**	**All locations**	**LLSB only**
All (Murmur + No-murmur) recordings	82.6, 89.4, 88.1%	88.4, 93.4, 92.7%
Murmurs only recordings	85.2, 93.4, 91.5%	**93.9, 93.7, 93.8%**

Note the best performance (shown in bold) was obtained when classifying murmur-only LLSB recordings.

### Test set results

**Figure 3:**
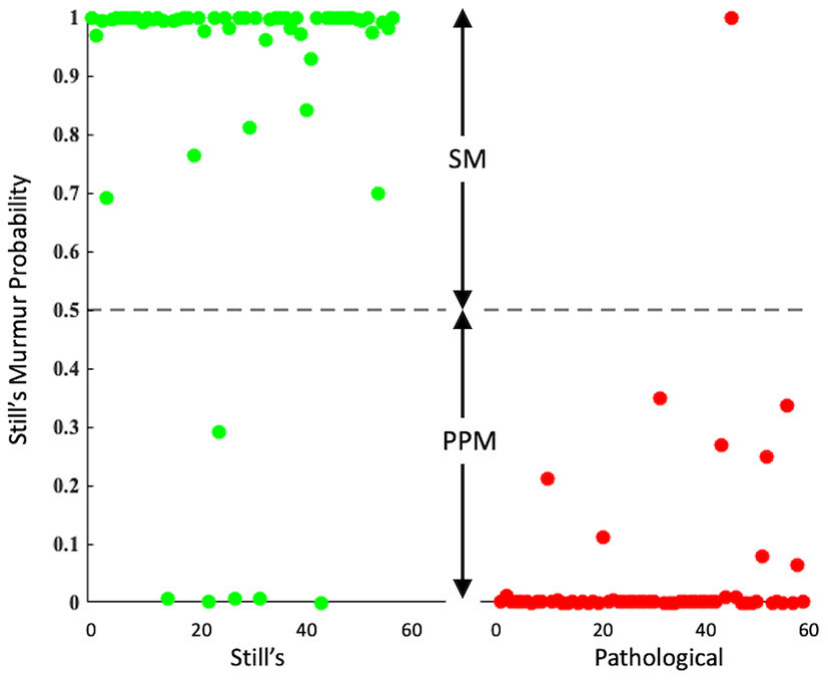
Scatter plot showing the classification of known Still's murmurs (in green) and known pathological murmurs (in red) in the test set. The algorithm classified the murmurs above the dashed line as SM and below it as PPM.

The classification result on the test set is presented in Figure 3. The horizontal axis depicts the clinically confirmed groups of 60 Still's murmurs (green) and 60 pathological murmurs (red). The vertical axis represents the probability of a recording being Still's murmur. The figure also shows the default decision threshold (0.5) separating the SM and PPM classes. All but 14 recordings in the test set were successfully segmented. When a recording fails segmentation, it is automatically assigned a probability value of 0, and is classified as PPM. This could occur, for example, with a holosystolic murmur or a continuous one. Thirteen of 60 pathological murmur recordings failed segmentation and were directly and correctly classified as PPM. Of the 60 Still's murmur recordings, one failed segmentation and, consequently, was misclassified as PPM.

The algorithm produced 54 true positives, 1 false positive, 59 true negatives, and 6 false negatives. These translate to the algorithm achieving a sensitivity of 90.0% (54 of 60 Still's murmurs correctly identified) and specificity of 98.3% (59 of 60 pathological murmurs correctly identified). The corresponding accuracy was 94.2%. When the recordings that failed segmentation were excluded from the calculation of performance parameters, the sensitivity was 91.5% (54 of 59 Still's murmurs correctly identified), the specificity was 97.9% (46 of 47 pathological murmurs correctly identified), and the overall accuracy was 94.3%. As is evident, the effect of excluding failed segmentation cases was a slight gain in sensitivity coupled with a slight loss in specificity.

**Figure 4:**
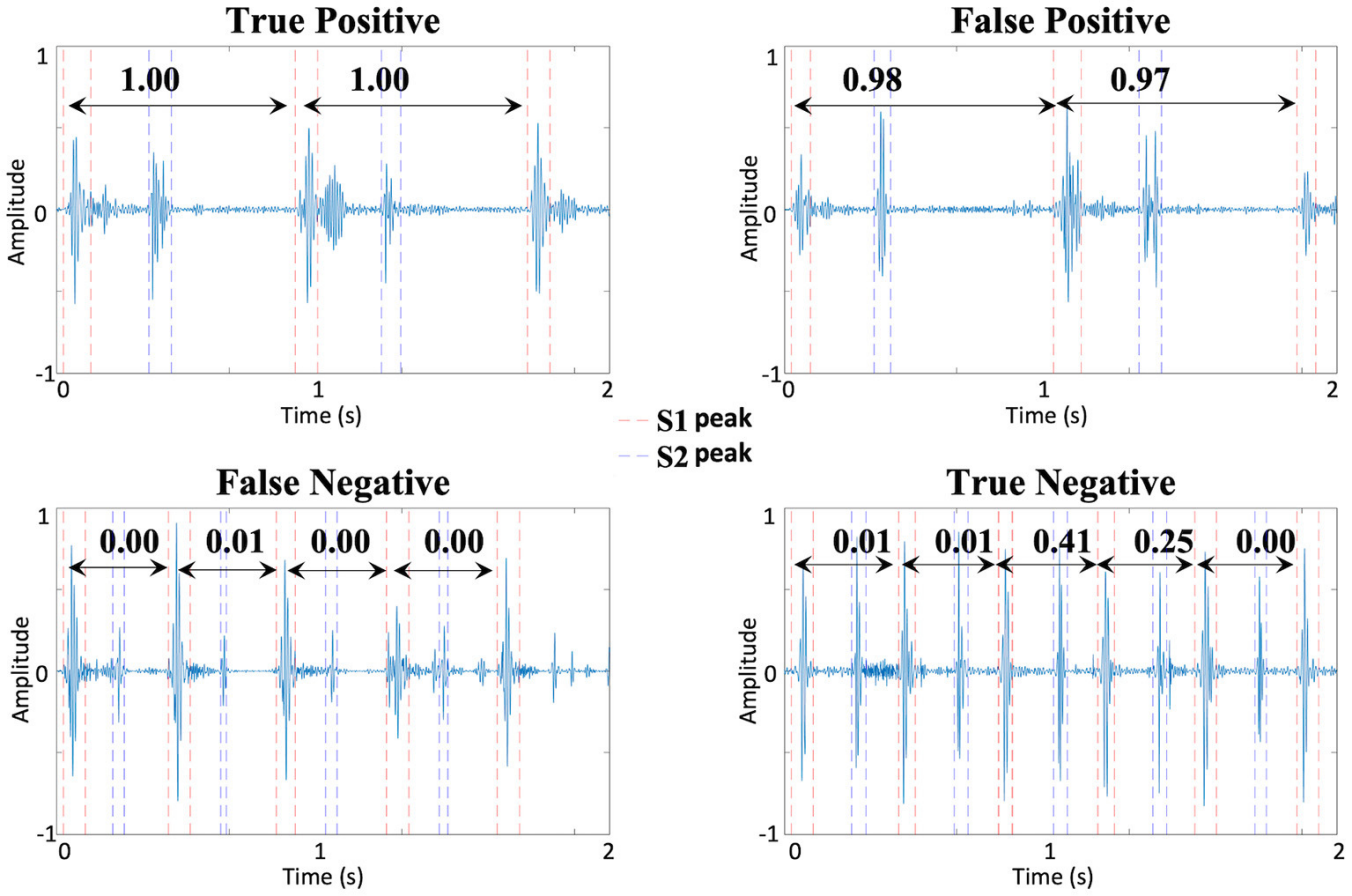
Representative true positive, false positive, true negative, and false negative examples with segmented S1 and S2 sounds and the probabilities of each cardiac cycle being a Still's murmur.

Figure 4 shows representative true positive, false positive, true negative and false negative recordings, with the probabilities of each individual cardiac cycle containing a Still's murmur. Among the six false negatives, i.e., Still's murmurs that were identified as PPM, one was due to segmentation failure and five others were misclassified after successful segmentation. When the segmentation failure case was inspected, it was observed that the recording had excessive noise. The other five Still's murmurs had been labeled as atypical by the cardiologist. The one false positive case was a recording of mild tricuspid regurgitation with murmur signal similar to that of a Still's murmur, albeit slightly higher pitched.

**Figure 5:**
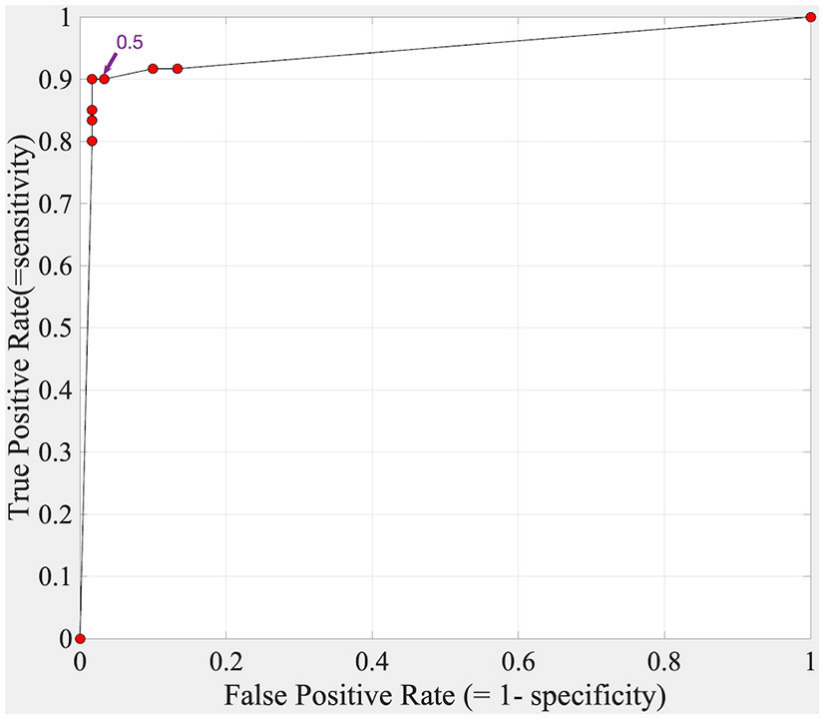
ROC curve for the Still's murmur identification algorithm tested on the test set.

We then studied the effect of varying the decision threshold; the corresponding ROC curve is shown in Figure 5. The AUC was 0.943. Note the default decision threshold of 0.5 was set a priori, and the CNN was trained using this threshold.

## Discussion

Our goal was to develop an algorithm that could aid PCPs in identifying Still's murmur as benign at the point of care to reduce over-referral of children with normal hearts, without eliminating those with true heart defects. The need for auscultation aimed at differentiating between innocent and pathological murmurs, as opposed to aiming at specific cardiac diagnoses, is central to the current PCP practice model in the United States (34).

In our implementation, the highest identification accuracy was achieved when the algorithm was trained and tested utilizing LLSB-only murmur recordings. The comparative analysis informed the design of our second experiment, testing the algorithm on an independent test set of echocardiography-confirmed murmurs. This “LLSB-and murmurs-only” strategy is appropriate for the intended clinical application, as LLSB is the most common location at which PCPs consistently examine the heart, and limiting the recording to a single location minimizes acquisition time (35).

The deep-learning approach presented here was slightly less accurate than our previously reported SVM and ANN classification methods (AUC of 0.943 vs. 0.966) (18). This trend is reflected in sensitivity (90 vs. 84–93%) and specificity (98.3 vs. 91–99%) figures as well. Our previous study used a significantly smaller dataset, hand-crafted features, and employed leave-one-out strategy for testing the algorithm. The present study took a more rigorous and reproducible approach involving a larger dataset and an independent test set. As with most applications, we believe deep learning is a superior approach in the long term as it is not based on manually identified features. More importantly, we believe that deep learning will have a higher performance ceiling compared with SVM and ANN methods.

Deep learning is data-intensive; our approach of performing the classification on individual cardiac cycles allowed us to have a dataset sufficient in size for training and testing the CNN. Because murmur characteristics vary beat-to-beat, we believe that this approach is not only desirable from a deep-learning perspective but also helps to achieve robust classification. Murmur libraries, some open-access, exist, but they are small and diverse in disease distribution and patient characteristics, and do not focus on pediatric murmurs (36, 37). It may be possible to utilize them in the future for training a learning-based algorithm, but this task remains difficult and of limited value at present.

We defined a positive result as indicative of Still's murmur and a negative result as indicative of a potentially pathological murmur. In general, clinicians think of sensitivity as correct identification of pathology, as opposed to non-pathology. But since the purpose of our algorithm is to identify non-pathological (i.e., obvious Still's) murmurs, and weed out a significant proportion of them, the terms sensitivity and specificity have been applied with their standard definitions. Therefore, to compare our results to traditionally reported classification performance numbers, the terms “sensitivity” and “specificity” must be reversed. A direct comparison with other methods also needs to take in account that the PPM class includes innocent murmurs that are not Still's. The proportion of this group of murmurs is significantly smaller than that of Still's murmurs in both incidence and those referred to pediatric cardiologists (1).

The notable prior studies on pediatric heart murmur classification have reported varied approaches, numbers of recordings, and results. Pretorius et al. reported a sensitivity of 91% and specificity of 94% in identification of pathological murmurs utilizing an ensemble ANN classifier and using recordings from 381 patients, from one of four chest locations (23). In another study, Pyles et al. reported 78.5% sensitivity and 92.6% specificity in identifying pathological murmurs from 149 subjects (21). Evaluation of a commercially available computer-aided auscultation software program on 126 subjects yielded a sensitivity of 83.9% and a very low specificity of 30.3% in identification of pathological murmurs against echocardiography (38). More recently, Thompson et al. reported achieving 93% sensitivity and 81% specificity for identifying pathological cases in a comparatively larger set of recordings from 603 predominantly pediatric patients (39). These studies focus on detection of pathological murmurs with the goal of maximizing sensitivity. By focusing on Still's murmur, our algorithm takes a different approach. Nevertheless, our algorithm incorrectly labeled only one pathological murmur (mild tricuspid regurgitation), and that was one of questionable clinical import and unlikely to alter management.

In the future, going beyond a binary classification may be possible, but, at this point, the losses in sensitivity and specificity would outweigh the benefits of adding other innocent murmurs, as Still's murmur is by far the largest cause of pediatric cardiology over-referrals. We can only speculate as to why that is, but we believe it is that most primary care providers caring for children auscultate chiefly—or perhaps only—over the precordium. The other two common innocent murmurs between ages 2 and 18 are the venous hum, which is heard at the right upper sternal border to the mid-right clavicle, and the innocent right ventricular outflow murmur often heard in older children, at the left upper sternal border, usually only supine. Atypical Still's murmurs will be classified by the algorithm as PPM. Our goal is not to remove all Still's murmurs from the referral cases, but rather to cull out the obvious, typical ones. This should have a substantial impact on referral patterns and use of resources.

Our algorithm's 90% sensitivity is high enough to ensure that the vast majority of Still's murmurs will be correctly recognized. As a decision support system, it could have a considerable impact on clinical practice by reducing the currently high rate of Still's murmur referrals made to pediatric cardiologists. In this context, specificity is critically important so as to minimize false negatives which could have negative clinical consequences. We note that the adoption of mandated critical congenital heart disease (CCHD) screening in the newborn in the United States substantially reduces the stakes in misclassifying a pathological murmur, as this screening selects out the vast majority of serious congenital heart defects before discharge home from the birth hospital (40).

Our approach would not identify some lesions such as atrial septal defects (ASDs), which are among the latest-diagnosed congenital cardiac heart defects in the current practice model, often missed until adulthood. This delay in diagnosis rarely, if ever, causes harm. Our approach might also be less accurate for cardiac murmurs due to acquired heart disease (e.g., rheumatic fever) and to those structural heart defects presenting later in childhood (e.g., discrete subaortic stenosis). Given the low incidence of such lesions in the U.S. population, we did not evaluate the accuracy of our algorithm in these settings. Subjects <2 years were not included in this study. In this age group, given the high sensitivity of newborn CCHD screening, critical congenital heart disease very rarely presents with a murmur in an asymptomatic child.

In conclusion, Still's murmur is responsible for a large over-referral problem in the United States. We have developed a deep-learning algorithm capable of identifying this murmur with high accuracy. The algorithm has the potential to serve as decision support in reducing the rate of unnecessary pediatric cardiologist referrals and use of echocardiography for a common benign finding. The future directions of our study include additional data acquisition, algorithmic improvements, prospective testing of the algorithm on recordings made in pediatric cardiology clinics against the findings of pediatric cardiologists, and the demonstration of acceptability of this new model to the PCP population.

## Data availability statement

The datasets presented in this article are not readily available because they are subject to intellectual property protection. Requests to access the datasets should be directed to rdorosho@childrensnational.org.

## Ethics statement

The studies involving human participants were reviewed and approved by Children's National Hospital IRB. Written informed consent from the participants' legal guardian/next of kin was not required to participate in this study in accordance with the national legislation and the institutional requirements.

## Author contributions

RS conceptualized, designed and directed the study, drafted the initial manuscript, and revised and prepared the final manuscript. GV performed data processing, trained and tested the neural network models, and assisted with generating results. TJ and JI assisted with organizing the study data, data processing, and drafting and review of the manuscript. YA assisted with the revision of the manuscript. RWD conceptualized, designed and directed the study, designed data collection, collected the study data, and critically reviewed and revised the manuscript. All authors approved the final manuscript as submitted and agree to be accountable for all aspects of the work.

## Funding

The study was supported by the NIH Grant R42HL131081.

## Conflict of interest

Authors RS and RWD are founders of AusculTech Dx. Authors GV, TJ, and JI are employees of AusculTech Dx. The remaining authors declare that the research was conducted in the absence of any commercial or financial relationships that could be construed as a potential conflict of interest.

## Publisher's note

All claims expressed in this article are solely those of the authors and do not necessarily represent those of their affiliated organizations, or those of the publisher, the editors and the reviewers. Any product that may be evaluated in this article, or claim that may be made by its manufacturer, is not guaranteed or endorsed by the publisher.

## Abbreviations

ANN: artificial neural network
AUC: area under the curve
CNN: convolutional neural network
LLSB: lower left sternal border
PCP: primary care provider
SM: classified as Still's murmur
PPM: classified as potentially pathological murmur
ROC: receiver operating characteristic.
